# A Minister of Public Health

**Published:** 1910-07-16

**Authors:** 


### A Minister of Public Health.

The importance of securing a central Public Health Authority that should control the various Departments which are now partly under one member of the Cabinet and partly under another, and thereby effect a saving of time and money, with, as an added advantage, the creation of a visible and permanent head to which the public might appeal in cases of doubt, has often been advocated in these pages. We believe that the medical profession will cordially welcome the establishment of such a special post, provided it is given to one whose integrity and standing merit the absolute confidence of both hospital and medical men.

We do not think that, in the discussion of this important subject, any valid arguments against the creation of a special post, that of Minister of Public Health, have been advocated by those who do not care for the centralisation of authority, and who believe, honestly enough, that our present position is good enough as it is, and that no further advantage will be gained by extending the already wide scope of the medical officer to the Local Government Board. The main contention against the theory that a Minister of Public Health is necessary is the argument that it will be extremely difficult to assign to such an official a list of duties and responsibilities which will satisfy everyone, will not clash with those appertaining to other departments, and will really tend to secure harmonious co-operation between the institutions, the profession, and the State. This argument, it is evident, is largely based on the admittedly difficult problem of arranging the numerous responsibilities which medical officers of health, superintendents, secretaries, educational medical authorities and others at present have to take into account. No Minister, it is safe to say, will have a wider range of activity than the man who is to preside over the Department of Public Health. His position will be no sinecure; his duties will be onerous and many.

Hitherto this creation of a special health department under Government has been a pious hope, chiefly because in no English-speaking country has a central authority of the kind we advocate as yet been established. It is true an analogy is to be found in foreign States; but Englishmen characteristically dislike imitating a foreign example. Now, however, the United States has made the attempt, and a Bill for the establishment of a Department of Public Health has actually been introduced into the Senate, and appears to have met with such general support that it is only a question of time before the first Minister of Public Health is actually appointed in America. It is interesting to scan the provisions of this Bill, which suggests the establishment of a special department, under the supervision of a Secretary, who shall be a Cabinet officer, appointed by the President, at a salary of £2,500 per annum, and who shall have under his control all "departments and bureaus, excepting the Department of War and of the Navy, affecting the medical, surgical, biological, or sanitary service, or any questions relative thereto." In amalgamating with the more purely medical and professional subjects, the biological and scientific side of public health—the bureau of entomology, of chemistry, and of veterinary science, for instance—the promoters of the American Bill go logically much further than we in England are likely to do. The Bill has been cordially endorsed by President Taft, whose interest in what may be called the educational side of medicine has always been great. In the United States, as here in England, there exist various Government departments which now meddle—there is no other word for it—with matters of public health to mutual disadvantage and with a tendency towards stultifying themselves in the eyes of a discriminating public. With us the State is even more active in meddling, in a cursory, and therefore extremely demoralising, manner, in matters in which it ought to take an active, vigorous, and thoroughly practical interest. A perusal of the American draft Bill is to be commended to every medical man, every institutional worker, and every public man who is in any way concerned in the welfare of the nation and the maintenance of public sanity. It is for them to bring its provisions to the attention of the legislators, until we too have the chance of getting a Minister of Public Health of our own.

